# Initial reduction of the primary tumor or lymph nodes: which is the better prognostic factor in patients with esophageal squamous cell carcinoma receiving neoadjuvant chemotherapy followed by surgery?

**DOI:** 10.1007/s10388-025-01128-5

**Published:** 2025-04-28

**Authors:** Takaomi Hagi, Osamu Shiraishi, Masuhiro Terada, Atsushi Yamada, Masashi Kohda, Tomoya Nakanishi, Yoko Hiraki, Hiroaki Kato, Atsushi Yasuda, Masayuki Shinkai, Motohiro Imano, Takushi Yasuda

**Affiliations:** Department of Surgery, Kindai University Faculty of Medicine, 377-2 Ohno-higashi, Osaka-Sayama, Osaka 589-8511 Japan

**Keywords:** Neoadjuvant chemotherapy, DCF, Esophageal cancer, Initial tumor reduction, Lymph node metastasis, Prognostic factor

## Abstract

**Background:**

Early response of the primary tumor (PT) to neoadjuvant chemotherapy (NAC) in patients with esophageal squamous cell carcinoma (ESCC) is considered a potential predictor of postoperative prognosis. However, the role of metastatic lymph nodes (LNs) remains poorly understood. This study aimed to compare the predictive value of early response in PT and LNs for postoperative prognosis.

**Methods:**

We enrolled 124 consecutive patients who received NAC-docetaxel, cisplatin, 5-fluorouracil (DCF) followed by surgery for ESCC between April 2010 and March 2020. Initial tumor reduction of the PT (ITR-PT) and LN (ITR-LN), defined as the percentage decrease in tumor shorter diameter after the first course of NAC-DCF, was evaluated using computed tomography. The optimal cut-off values of ITRs were determined using receiver operating characteristic curves and Cox regression models, and their relationship with recurrence-free survival (RFS) was analyzed.

**Results:**

The median ITR-PT and ITR-LN were 21.77% and −0.88%, respectively. The optimal cut-off values for predicting prognosis were approximately 10% for ITR-PT (hazard ratio [HR], 3.23; 95% confidence interval [CI], 1.84–5.64) and −10% for ITR-LN (HR, 2.20; 95% CI, 1.27–3.80). ITR-PT showed a greater impact on RFS (3-year RFS: ITR-PT ≥ 10%, 66.1%; ITR-PT < 10%, 18.4%; log-rank *P* < 0.001) compared with ITR-LN (3-year RFS: ITR-LN ≥ −10%, 64.1%; ITR-PT < −10%, 34.3%; log-rank *P* = 0.004). Multivariate analysis of RFS identified ypN, ITR-PT, and ITR-LN as independent prognostic factors.

**Conclusions:**

Both ITR-PT and ITR-LN are promising predictors of survival in patients with ESCC who underwent NAC-DCF plus surgery. ITR-PT may be a stronger prognostic factor than ITR-LN.

**Supplementary Information:**

The online version contains supplementary material available at 10.1007/s10388-025-01128-5.

## Introduction

Esophageal cancer is the seventh most common cancer worldwide [1]. Neoadjuvant chemotherapy (NAC) plus surgery is the standard treatment strategy for eliminating micrometastases and increasing resectability [2–5]. Recently, a triplet NAC regimen with a combination of docetaxel, cisplatin, and 5-fluorouracil (NAC-DCF) showed a survival benefit compared with doublet chemotherapy (cisplatin and 5-fluorouracil) and has become the standard treatment for locally advanced esophageal squamous cell carcinoma (ESCC) in Japan [6]. Although this regimen is highly effective and improves survival in many patients, some are refractory to treatment and have poor prognoses after surgery [7]. Early identification of NAC-DCF non-responders may be beneficial in deciding whether to discontinue or change treatment.

Lymph node (LN) metastases are more strongly associated with poor prognosis in patients with ESCC compared with the progression of the primary tumor (PT) [8, 9]. Additionally, in patients with ESCC who receive NAC plus surgery, the response of LNs to NAC is a better predictor of postoperative survival than the response to PT, as observed in both clinical and pathological examinations [10, 11]. We previously reported that initial tumor reduction of the PT (ITR-PT), defined as the response to PT after the first course of NAC-DCF, was a good predictor of survival in patients with ESCC who received NAC-DCF followed by surgery [12]. However, this assessment method focuses solely on the reduction in PT in response to NAC-DCF and neglects the reduction in metastatic LNs. Based on the above, the early response of metastatic LNs to NAC (i.e., the initial tumor reduction of LNs [ITR-LN]) may potentiate a better prognostic factor than ITR-PT.

In the present study, we aimed to investigate the optimal assessment method for ITR-LN that best correlated with postoperative survival in patients with ESCC who received NAC-DCF plus surgery. In addition, the optimal cutoff values of ITR-PT and ITR-LN for predicting prognosis were estimated and compared to determine which of these two parameters was a better predictor of postoperative survival.

## Methods

### Patients

The eligible population in this study consisted of consecutive patients with histologically diagnosed ESCC treated with NAC-DCF followed by esophagectomy without distant metastases (excluding supraclavicular LN metastasis) between April 2010 and March 2020 at the Department of Surgery, Kindai University Hospital, Osaka-Sayama, Japan. The following patients were excluded from this study: patients with cervical esophageal and esophagogastric junction cancer, patients who failed to accomplish two or three courses of NAC-DCF, patients who underwent macroscopic non-curative resection, and patients without detailed medical records, including follow-up computed tomography (CT) scans before and after every course of NAC-DCF. Patients with unmeasurable PT or clinical-N (cN) negativity on baseline CT were also excluded. All patients were staged according to the Eighth Edition of the Union for International Cancer Control TNM classification [13]. Tumor responses to chemotherapy were classified according to the Japanese Classification of Esophageal Cancer (11 th edition) [14, 15]. This study was approved by the Institutional Review Board of Kindai University Hospital (No. 2024–10), and the need for written informed consent was waived.

### Treatment

The standard NAC-DCF regimen included an intravenous dose of docetaxel (70 mg/m^2^) and cisplatin (70 mg/m^2^) administered on day 1, accompanied by a continuous intravenous infusion of 5-fluorouracil (700 mg/m^2^) from days 1 to 5. This treatment cycle was repeated every 3 weeks for a total of two courses. However, in certain cases, patients received a third course of the DCF regimen. This modification was implemented because our institution participated in a multicenter randomized phase II clinical trial that compared the effectiveness of two versus three courses of NAC-DCF in treating locally advanced ESCC [16, 17]. Consequently, the decision to administer three courses was made without any selection bias. Upon completion of the NAC-DCF regimen, all patients underwent a McKeown esophagectomy with either two- or three-field lymphadenectomy, following the guidelines established by the Japan Esophageal Society (2022) [18].

### Computed tomography evaluation of tumor reduction

CT scans for tumor evaluation were performed before starting NAC-DCF and 2–3 weeks after the first day of each course, using contrast-enhanced CT with a slice thickness of ≤ 2.5 mm. Tumor reduction was evaluated based on the previously reported criteria [12], measuring the longer and shorter PT and LN diameters on axial slices of the largest tumor section. A shorter diameter was defined as the maximum tumor measurement perpendicular to the longest axis diameter in both the PT and LNs. For both PT and LNs, tumor reduction was defined as the percentage decrease in the shorter diameter of the tumor 2–3 weeks after the first day of NAC-DCF in each course compared with the baseline measurement. The assessment was conducted by an investigator blinded to the clinical data.

Considering PT, a tumor with a long-axis diameter of < 20 mm on baseline CT was deemed unmeasurable, as previously reported [12]. The reduction in PT during the first course of NAC-DCF was regarded as ITR-PT. As for LNs, based on the Japanese Classification of Esophageal Cancer (12 th edition) [19, 20], nodes with a shorter diameter of ≥ 6 mm on baseline CT were considered metastatic LNs. As metastatic LNs often exist in multiple numbers, the reduction of metastatic LNs for each case was assessed using the following four assessment methods: (1) worst-LN reduction, the reduction rate of the metastatic LN with the poorest response; (2) best-LN reduction, the reduction rate of the metastatic LN with the best response; (3) average-LN reduction, the average reduction rate of all metastatic LNs; and (4) sum-LN reduction, the reduction rate of a sum of the shorter diameters for all metastatic LNs in accordance with the Response Evaluation Criteria in Solid Tumours (RECIST) criteria [21]. Among these four methods, the one that showed the strongest relationship with prognosis was defined as the reduction in LNs, with the reduction in LNs during the first course of NAC-DCF regarded as the ITR-LN.

### Statistical analyses

A receiver operating characteristic (ROC) curve was generated to assess the optimal tumor reduction threshold for prognosis. The associations between clinicopathological factors and ITRs were examined using the chi-squared test for categorical variables and the Mann–Whitney *U* test for continuous variables. Recurrence-free survival (RFS) was defined as the time from surgery to recurrence or death from any cause, whereas overall survival (OS) was defined as the time from surgery to death from any cause. Kaplan–Meier survival curves were constructed to estimate RFS and OS, and comparisons were made using the log-rank test. A Cox proportional hazards regression model employing stepwise selection was used to determine the optimal tumor reduction cut-off. Variables that showed associations with RFS (*P* < 0.1) in the univariate analyses were subsequently analyzed using a multivariate Cox proportional hazards model. Statistical significance was defined as a *P* value of < 0.05. All statistical analyses were performed using the IBM SPSS Statistics software (version 22.0; IBM Corporation, Armonk, NY, USA).

## Results

### Patient characteristics

Of the 230 patients with ESCC treated with NAC-DCF followed by esophagectomy, 39 were excluded (15 with cervical esophagus and esophagogastric junction cancer, 13 who failed to accomplish two or three courses of NAC-DCF owing to adverse events, two underwent macroscopic non-curative resection, and nine without detailed medical records, including follow-up CT scans before and after every course of NAC-DCF). In the remaining 191 patients, the PT and LNs were measured using baseline CT. After excluding 37 patients with unmeasurable PT and 30 patients with cN-negative PT, 124 patients were included in the analysis. Background data of the 124 eligible patients are shown in Table 1. The median length of baseline shorter diameter of the primary tumor was 22.7 mm (range, 11.3–39.3 mm), and the median number of metastatic LNs at baseline was 3 (range, 1–9). Two courses of NAC-DCF were administered to 101 (81.5%) patients, and 23 (18.5%) patients received three courses.

**Table 1:** Clinicopathological characteristics

	n = 124	(%)
Age in years		
Median (range)	67 (41–79)	
Sex		
Male	103	83.1
Female	21	16.9
Location		
Ut	23	18.5
Mt	55	44.4
Lt	46	37.1
Histological differentiation (squamous cell carcinoma)		
Well	14	11.3
Moderately	47	37.9
Poorly	24	19.4
Unknown	39	31.5
Baseline shorter diameter of the primary tumor (mm)		
Median (range)	22.7 (11.3–39.3)	
Number of metastatic lymph nodes at baseline		
Median (range)	3 (1–9)	
Baseline shorter diameter of metastatic lymph nodes (mm)		
Median (range)	12.19 (6.41–40.55)	
cT		
1	2	1.6
2	19	15.3
3	99	79.8
4	4	3.2
cN		
1	36	29.0
2	82	66.1
3	6	4.8
cM^a^		
0	110	88.7
1	14	11.3
cStage		
II	16	12.9
III	89	71.8
IV	19	15.3
Number of NAC-DCF courses		
2	101	81.5
3	23	18.5
ypT		
0	12	9.7
1	31	25.0
2	20	16.1
3	58	46.8
4	3	2.4
ypN		
0	41	33.1
1	45	36.3
2	27	21.8
3	11	8.9
ypM^a^		
0	110	88.7
1	14	11.3
ypStage		
0	9	7.3
I	11	8.9
II	38	30.6
III	44	35.5
IV	22	17.7
Residual tumor		
R0	118	95.2
R1	6	4.8
Lymphatic invasion		
Negative	79	63.7
Positive	40	32.3
Unknown	5	4.0
Vascular invasion		
Negative	101	81.5
Positive	18	14.5
Unknown	5	4.0

The percentage may not add up to 100 due to rounding. Clinical and pathological stage was according to the Eighth Edition of the Union for International Cancer Control TNM classification

*Ut* upper thorax, *Mt* middle thorax, *Lt* lower thorax, *NAC-DCF* neoadjuvant chemotherapy with the combination of docetaxel, cisplatin, and 5-fluorouracil

^a^Includes supraclavicular LN metastasis

### Optimal assessment method and cut-off values of initial tumor reduction (ITRs)

**Fig. 1:**
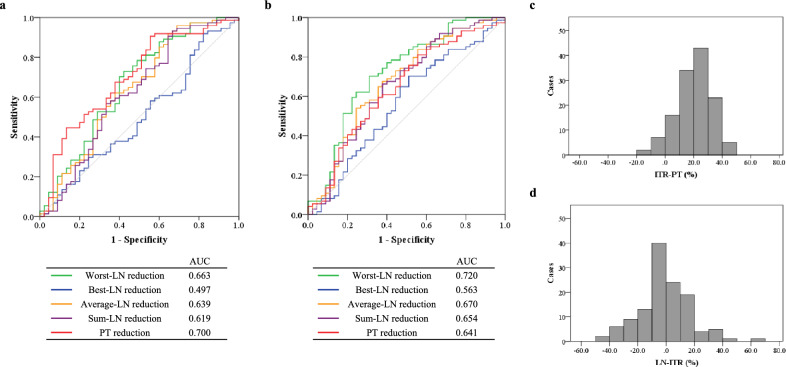
Receiver operating characteristic (ROC) curves for the assessment of 2-year recurrence-free survival and distribution of initial tumor reductions (ITRs). ROC curves based on the reduction of metastatic lymph nodes (LNs), assessed by four different methods, and the primary tumor (PT) **a** during the first course and **b** after all courses of neoadjuvant chemotherapy regimen with the combination of docetaxel, cisplatin, and 5-fluorouracil. Green, blue, yellow, purple, and red curves indicate the worst-LN reduction, best-LN reduction, average-LN reduction, sum-LN reduction, and PT reduction, respectively. Gray line indicates the diagonal reference line. AUC indicates the area under the ROC curve. Histogram of **c** ITR-PT and **d** ITR-LN in all cases

For tumor reduction during the first course, the area under the ROC curve (AUC) for PT reduction (AUC, 0.700; standard error [SE], 0.050; *P* < 0.001; 95% confidence interval [CI], 0.601–0.798) was larger than that of any other methods for assessing LN reduction (Fig. 1a). Additionally, the AUC for the worst-LN reduction (AUC, 0.663; SE, 0.053; *P* = 0.003; 95% CI, 0.559–0.768) was the largest among all the methods for assessing LN reduction. For tumor reduction after all courses, the AUC for the worst-LN reduction (AUC, 0.720; SE, 0.051; *P* < 0.001; 95% CI, 0.621–0.819) was the largest among all methods for assessing LN and PT reductions (Fig. 1b). Therefore, the worst-LN reduction during the first course of NAC-DCF was defined as the ITR-LN. Based on the Youden index analysis, the optimal cut-off values for predicting prognosis were 11.61% for ITR-PT (sensitivity 90.5%, specificity 44.4%) and −9.65% for ITR-LN (sensitivity 73.0%, specificity 57.8%). The median values were 21.77% (range −11.45–46.76%) for ITR-PT and –0.88% (range −46.45–69.89%) for ITR-LN (Fig. 1c and d).

Cox proportional hazards regression analyses for RFS, performed in increments of 10% tumor reduction, identified that a 10% ITR-PT (hazard ratio [HR], 3.23; 95% CI, 1.85–5.64; *P* < 0.001) and a −10% ITR-LN (HR, 2.20; 95% CI, 1.27–3.80; *P* = 0.005) were significant cut-off points with a notable impact on prognosis with relatively high ratio between upper and lower confidence intervals (Table 2). Based on these results, ITR-PT 10% and ITR-LN –10% were used as cut-off values for further analyses.

**Table 2:** Univariate logistic regression analysis of optimal cut-off value for initial tumor reduction of primary tumor and lymph node based on recurrence-free survival

Cut-off value	ITR-PT								
	40%	30%	20%	10%	0%	−10%	−20%	−30%	−40%
Number of patients									
Responder	5 (4.0%)	27 (21.8%)	70 (56.5%)	99 (79.8%)	115 (92.7%)	122 (98.4%)			
Non-responder	119 (96.0%)	97 (78.2%)	54 (43.5%)	25 (20.2%)	9 (7.3%)	2 (1.6%)			
RFS									
HR	0.77	3.90	2.23	3.23	3.38	3.68			
95% CI (*L*–*U*)	0.24–2.45	1.56–9.76	1.34–3.73	1.85–5.64	1.59–7.15	0.89–15.14			
* L/U* ratio	0.098	0.16	0.36	0.33	0.22	0.059			
* P* value	0.66	**0.004**	**0.002**	** < 0.001**	**0.001**	0.071			

Cut-off value	ITR-LN								
	40%	30%	20%	10%	0%	−10%	−20%	−30%	−40%
Number of patients									
Responder	2 (1.6%)	7 (5.6%)	11 (8.9%)	30 (24.2%)	54 (43.5%)	94 (75.8%)	107 (86.3%)	116 (93.5%)	122 (98.4%)
Non-responder	122 (98.4%)	117 (94.4%)	113 (91.1%)	94 (75.8%)	70 (56.5%)	30 (24.2%)	17 (13.7%)	8 (6.5%)	2 (1.6%)
RFS									
HR	20.83	1.95	2.27	1.57	1.66	2.20	2.17	1.97	4.17
95% CI (*L*–*U*)	–	0.48–8.00	0.71–7.25	0.82–3.03	0.99–2.81	1.27–3.80	1.17–4.22	0.77–5.03	0.55–31.25
* L/U* ratio	–	0.060	0.098	0.27	0.35	0.33	0.28	0.15	0.018
* P* value	0.41	0.35	0.17	0.18	0.057	**0.005**	**0.022**	0.15	0.17

Bold indicates significant difference

*ITR* initial tumor reduction, *PT* primary tumor, *RFS* recurrence-free survival, *HR* hazard ratio, *CI* confidence interval, *L* lower confidence limit, *U* upper confidence limit, *LN* lymph node

### Status of ITRs

The relationship between the clinicopathological characteristics and ITRs was analyzed (Table 3). ITR-PT < 10% was observed in 25 (20.2%) patients, and ITR-LN < −10% was observed in 30 (24.2%) patients. Patients with ITR-PT < 10% had a significantly higher proportion of ypM1 (supraclavicular LN metastasis) (*P* = 0.025), were positive in vascular invasion (*P* = 0.032), and were clinical non-responder to NAC-DCF (*P* < 0.001). Additionally, patients with ITR-LN < −10% had a significantly higher proportion of ypN2–3 (*P* < 0.001) and were pathological non-responder to NAC-DCF (*P* = 0.033).

**Table 3:** Clinicopathological characteristics according to initial tumor reduction of primary tumor and lymph node

	ITR-PT		*P* value	ITR-LN		*P* value
	< 10% (n = 25, %)	≥ 10% (n = 99, %)		< –10% (n = 30, %)	≥ –10% (n = 94, %)	
Age in years			0.21			0.89
Median	70	67		67	67	
Range	56–76	41–79		41–78	41–79	
Sex			0.46			0.55
Male	22 (88.0%)	81 (81.8%)		26 (86.7%)	77 (81.9%)	
Female	3 (12.0%)	18 (18.2%)		4 (13.3%)	17 (18.1%)	
Location			0.71			0.76
Ut	4 (16.0%)	19 (16.0%)		5 (16.7%)	18 (19.1%)	
Mt/Lt	21 (84.0%)	80 (84.0%)		25 (83.3%)	76 (80.9%)	
cT			0.10			0.25
1–2	7 (28.0%)	14 (14.1%)		3 (10.0%)	18 (19.1%)	
3–4	18 (72.0%)	85 (85.9%)		27 (90.0%)	76 (80.9%)	
cN			0.18			**0.016**
1	11 (44.0%)	25 (25.3%)		3 (10.0%)	33 (35.1%)	
2–3	14 (56.0%)	74 (74.7%)		27 (90.0%)	61 (64.9%)	
cM^a^			0.90			0.11
0	22 (88.0%)	88 (88.0%)		29 (96.7%)	81 (86.2%)	
1	3 (12.0%)	11 (11.1%)		1 (3.3%)	13 (13.8%)	
ypT			0.45			0.17
0–2	11 (44.0%)	52 (52.5%)		12 (40.0%)	51 (54.3%)	
3–4	14 (56.0%)	47 (47.5%)		18 (60.0%)	43 (45.7%)	
ypN			0.26			** < 0.001**
0–1	15 (60.0%)	71 (71.7%)		12 (40.0%)	74 (78.7%)	
2–3	10 (40.0%)	28 (28.3%)		18 (60.0%)	20 (21.3%)	
ypM^a^			**0.025**			0.69
0	19 (76.0%)	91 (91.9%)		26 (86.7%)	84 (89.4%)	
1	6 (24.0%)	8 (8.1%)		4 (13.3%)	10 (10.6%)	
Residual tumor			0.83			0.66
R0	24 (96.0%)	94 (94.9%)		29 (96.7%)	89 (94.7%)	
R1	1 (4.0%)	5 (5.1%)		1 (3.3%)	5 (5.3%)	
Lymphatic invasion^b^			0.057			0.080
Negative	12 (50.0%)	67 (70.5%)		16 (53.3%)	63 (70.8%)	
Positive	12 (50.0%)	28 (29.5%)		14 (46.7%)	26 (29.2%)	
Vascular invasion^b^			**0.032**			0.79
Negative	17 (70.8%)	84 (88.4%)		25 (83.3%)	76 (85.4%)	
Positive	7 (29.2%)	11 (11.6%)		5 (16.7%)	13 (14.6%)	
Clinical response^c^			** < 0.001**			0.10
Responder	10 (40.0%)	79 (79.8%)		18 (60.0%)	71 (75.5%)	
Non-responder	15 (60.0%)	20 (20.2%)		12 (40.0%)	23 (24.5%)	
Pathological regression^d^			0.57			**0.033**
Responder	7 (31.8%)	36 (38.3%)		5 (19.2%)	38 (42.2%)	
Non-responder	15 (68.2%)	58 (61.7%)		21 (80.8%)	52 (57.8%)	

Responder was defined as grade III or IV, and non-responder as grade I or II

Bold indicates significant difference

*ITR* initial tumor reduction, *PT* primary tumor, *LN* lymph node, *Ut* upper thorax, *Mt* middle thorax, *Lt* lower thorax

^a^Includes supraclavicular LN metastasis

^b^Unknown in 5 patients

^c^Responder was defined as complete or partial response, and non-responder as stable or progressive disease

^d^Unknown in 9 patients

### Long-term outcomes

**Fig. 2:**
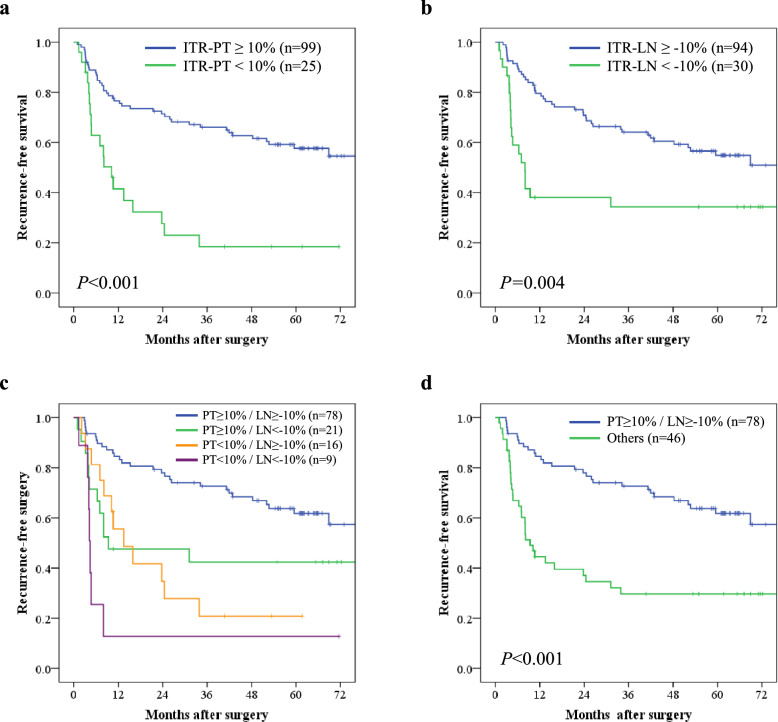
Kaplan–Meier recurrence-free survival according to **a** the initial tumor reduction of the primary tumor (ITR-PT) ≥ 10% and the ITR-PT < 10% groups, **b** the initial tumor reduction of the lymph node (ITR-LN) ≥ −10% and the ITR-LN < −10% groups, and **c** PT ≥ 10%/LN ≥ −10%, PT ≥ 10%/LN < −10%, PT < 10%/LN ≥ −10%, and PT < 10%/LN < −10% groups, **d** PT ≥ 10%/LN ≥ − 10% and other groups

The median follow-up period for the censored patients was 61.8 months. The RFS in the ITR-PT < 10% group was significantly worse than that in the ITR-PT ≥ 10% group (log-rank *P* < 0.001) (Fig. 2a). The 3-year RFS rates were 18.4% in the ITR-PT < 10% group and 66.1% in the ITR-PT ≥ 10% group. Additionally, the RFS in the ITR-LN < −10% group was significantly worse than that in the ITR-LN ≥ −10% group (log-rank *P* = 0.004) (Fig. 2b). The 3-year RFS rates were 34.3% in the ITR-LN < −10% group and 64.1% in the ITR-LN ≥ −10% group. Additionally, the 3-year RFS rates of ITR-PT ≥ 10%/ITR-LN ≥ −10%, ITR-PT ≥ 10%/ITR-LN < −10%, ITR-PT < 10%/ITR-LN ≥ −10%, and ITR-PT < 10%/ITR-LN < −10% were 72.6%, 42.3%, 20.8%, and 12.7%, respectively (Fig. 2c). Analysis of OS according to the ITR-PT and ITR-LN status showed a trend similar to that of RFS (Supplementary Fig. S1).

To assess the prognostic value of ITR-PT and ITR-LN status independent of other confounding factors, a Cox multivariate analysis for RFS with four clinicopathological factors was performed (Table 4). The multivariate analysis identified ITR-PT (*P* = 0.008) and ITR-LN (*P* = 0.048) as independent prognostic factors, along with ypN (*P* < 0.001).

**Table 4:** Univariate and multivariate analysis of recurrence-free survival

	Univariate analysis		Multivariate analysis	
	HR (95% CI)	*P* value	HR (95% CI)	*P* value
Age (years)				
< 65	1	0.18		
≥ 65	1.46 (0.84–2.53)			
Sex				
Male	1.57 (0.75–3.32)	0.23		
Female	1			
Location				
Ut	1.31 (0.72—2.38)	0.38		
Mt/Lt	1			
Number of NAC-DCF courses				
2	1.24 (0.63–2.44)	0.54		
3	1			
ypT				
0–2	1	0.11		
3–4	1.51 (0.91–2.50)			
ypN				
0–1	1	** < 0.001**	1	** < 0.001**
2–3	3.91 (2.35–6.51)		3.25 (1.92–5.51)	
Residual tumor				
R0	1	0.25		
R1	1.83 (0.66–5.06)			
Clinical response^a^				
Responders	1	** < 0.001**	1	0.19
Non-responders	2.51 (1.51–4.17)		1.47 (0.82–2.64)	
Pathological regression grade^b^				
Responders	1	0.18		
Non-responders	1.47 (0.84–2.56)			
ITR-PT				
< 10%	3.23(1.85–5.64)	** < 0.001**	2.34(1.25–4.38)	**0.008**
≥ 10%	1		1	
ITR-LN				
< –10%	2.20 (1.27–3.80)	**0.005**	1.81 (1.03–3.12)	**0.048**
≥ –10%	1		1	

Bold indicates significant difference

*HR* hazard ratio, *CI* confidence interval, *Ut* upper thorax, *Mt* middle thorax, *Lt* lower thorax, *NAC-DCF* neoadjuvant chemotherapy with the combination of docetaxel, cisplatin, and 5-fluorouracil, *ITR* initial tumor reduction, *PT* primary tumor, *LN* lymph node

^a^Responder was defined as complete or partial response, and non-responder as stable or progressive disease

^b^Responder was defined as grade III and IV, and non-responder as grade I and II

Of the 124 patients, 49 (39.5%) had recurrences during follow-up. The ITR-PT < 10% group showed significantly higher recurrence rates in lymphogenous metastasis (52.0% vs. 18.2%, *P* < 0.001) and kidney metastasis (12.0% vs. 1.0%, *P* = 0.005) compared with the ITR-PT ≥ 10% group (Supplementary Table S1). Moreover, lymphogenous metastasis occurred significantly more frequently in the ITR-LN < –10% group (43.3%) than in the ITR-LN ≥ –10% group (19.1%) (*P* = 0.008).

## Discussion

This cohort study demonstrated that the worst-LN reduction and the reduction rate of the metastatic LN with the poorest response best correlated with the frequency of recurrence in patients with ESCC who received NAC-DCF followed by surgery. PT was a better predictor of postoperative survival than metastatic LNs in terms of tumor response after the first course of NAC-DCF. The Cox multivariate analysis demonstrated that both ITR-PT and ITR-LN were independent prognostic factors. Additionally, the recurrence of lymphogenous metastases was significantly more frequent in the lower response groups for both ITR-PT and ITR-LN compared with the higher response groups.

In practice, two or three courses of NAC-DCF are commonly administered as standard therapy for ESCC. Early prediction of the efficacy of NAC-DCF on survival could aid in deciding whether to discontinue or modify the approach. Alternative strategies, such as radiotherapy or immune checkpoint inhibitors, might provide a better prognosis for patients with esophageal cancer who show limited response to NAC-DCF. We demonstrated that 20% of overall cohort had ITR-PT < 10%, and 24% had ITR-LN < −10%, both of which were associated with worse prognosis. Since this study primarily focused on identifying patient groups who are non-responders to NAC-DCF, these proportions seem reasonable for consideration of changing treatment strategy in clinical practice. Several previous studies on esophageal cancer have investigated early response-based therapies, focusing on reducing PT [22, 23]. For ESCC with NAC followed by surgery, several studies have explored the relationship between the early response of the PT and prognosis [24, 25], including our study [12]. A previous study in patients with non-small cell lung carcinoma investigated the early response in the PT and metastatic LNs as prognostic predictors and demonstrated that both responses were significantly correlated with survival [26]. However, no studies have examined the utility of the early response of metastatic LNs as a predictor of prognosis, and it remains unclear which ITR (PT or metastatic LNs) better reflects prognosis in patients with ESCC. To the best of our knowledge, this study is the first to evaluate and compare the ITRs of both PT and metastatic LNs as independent prognostic factors in patients with ESCC who received NAC-DCF followed by surgery.

When assessing the rate of reduction in metastatic LNs, they often exist in multiple numbers, making it complex and challenging to define an evaluation method. In this study, we defined four different assessment methods for the reduction rate of metastatic LNs, including a method in accordance with the RECIST criteria for evaluating measurable lesions (sum-LN reduction). As the RECIST criteria, a reliable method for assessing tumor response to treatment used worldwide, typically correlates well with prognosis, it is expected that sum-LN reduction would provide a strong predictive value. However, the worst-LN reduction correlated better with the frequency of recurrence than the sum-LN reduction, both during the first course and after all courses of NAC-DCF. One possible explanation for this is that the RECIST criteria are not specifically designed for patients treated with NAC. As one of the major aims of NAC is to eliminate microscopic distant metastases, the response of all metastatic LNs, including the LN with the poorest treatment response, may be more important than the overall response of the LNs.

ROC analysis for recurrence indicated that PT reduction during the first course of NAC-DCF was initially the stronger prognostic predictor. However, metastatic LN reduction, particularly worst-LN reduction, became more significant after all courses. A previous study reported that LN response to NAC predicted long-term survival more accurately than PT response in patients with metastatic ESCC [10], which supports our findings. Additionally, multivariate analysis identified both ITR-PT and ypN as independent prognostic factors, with ypN being the strongest predictor of prognosis. However, the assessment of ypN can only be performed postoperatively, whereas ITR-PT can be evaluated after the first course of NAC-DCF, providing an opportunity to predict prognosis and potentially adjust treatment strategies. Although less powerful than ypN as a prognostic factor, ITR-PT remains a valuable prognostic marker and serves as an early predictive tool, making it clinically useful in practical settings.

This shift in prognostic significance, where PT reduction is initially more predictive but metastatic LN reduction becomes stronger after all courses of NAC-DCF, may be due to biological differences in chemosensitivity between the PT and metastatic LNs. In fact, the proportion of vascular invasion was significantly higher in patients with ITR-PT < 10%, but not in ITR-LN. A previous study reported that 26% of patients with ESCC exhibited an inconsistent response to NAC between the PT and metastatic LNs [27]. Although the exact reason for this remains unclear, we hypothesized that PT responds to DCF treatment prior to metastatic LNs, with the latter showing a delayed response.

This study has some limitations. First, the diagnosis of metastatic LNs using CT remains controversial. Some studies used a combination of CT and 18 F-fluoro-2-deoxy-D-glucose positron emission tomography or endoscopic ultrasonography for diagnosis [28, 29], whereas others used different LN diameter cutoffs [30, 31]. However, none of the methods are definitive, with sensitivity for detecting metastatic LNs ranging from 29 to 94% and specificity from 38 to 98% [32]. Therefore, we used the Japanese Classification of Esophageal Cancer (12 th edition) [19, 20], with reported sensitivity and specificity of 65%–82% and 40%–52%, respectively. Second, this single-institution retrospective study with a relatively small patient sample is prone to selection bias, which we mitigated by collecting data from consecutive patients. Third, the assessment of PT and LNs was conducted by a single blinded investigator, raising concerns about inter-examiner variability. However, previous research showed high PT assessment consistency (intraclass correlation coefficient 0.749 [12]), and we believe this extends to LN assessment in this study. Finally, this study only included patients who received the DCF regimen as NAC, excluding other regimens. Further research is needed to generalize these findings to ESCC patients treated with different NAC regimens.

In conclusion, the reduction rate of metastatic LN with the poorest response was the best predictor of postoperative survival in patients with ESCC who received NAC-DCF followed by surgery. Although both ITR-PT and ITR-LN were strong predictors of prognosis, ITR-PT predicted postoperative survival better than ITR-LN. Early assessment should focus on PT reduction during the first course of treatment.

## Supplementary Information

Below is the link to the electronic supplementary material.

Supplementary file1 (DOCX 93 KB)

## Data Availability

Raw data were generated at Kindai University. The data that support the findings of this study are available from the corresponding author, O. S, upon reasonable request.
