# Supplementary material for: Initial reduction of the primary tumor or lymph nodes: which is the better prognostic factor in patients with esophageal squamous cell carcinoma receiving neoadjuvant chemotherapy followed by surgery?
Source: Esophagus. 2025 Apr 28;22(3):398–409. doi: 10.1007/s10388-025-01128-5 (PMC12167354; doi:10.1007/s10388-025-01128-5)
Supplement: Supplementary file 1 — Supplementary file1 (DOCX 93 KB) [file 10388_2025_1128_MOESM1_ESM.docx]

**Supplementary Table S1.** Patterns of Recurrence

|  | ITR-PT | |  | ITR-LN | |  |
| --- | --- | --- | --- | --- | --- | --- |
|  | < 10%  (n = 25, %) | ≥ 10%  (n = 99, %) | *P* value | < –10%  (n = 30, %) | ≥ –10%  (n = 94, %) | *P* value |
| Any recurrence | 16 (64.0%) | 33 (33.3%) | **0.005** | 15 (50.0%) | 34 (36.2%) | 0.18 |
| Hematogenous | 7 (28.0%) | 21 (21.2%) | 0.47 | 9 (30.0%) | 19 (20.2%) | 0.26 |
| Lung | 0 (0.0%) | 12 (12.1%) | 0.067 | 4 (13.3%) | 8 (8.5%) | 0.44 |
| Liver | 1 (4.0%) | 7 (7.1%) | 0.58 | 3 (10.0%) | 5 (5.3%) | 0.36 |
| Bone | 3 (12.0%) | 4 (4.0%) | 0.12 | 2 (6.7%) | 5 (5.3%) | 0.78 |
| Kidney | 3 (12.0%) | 1 (1.0%) | **0.005** | 1 (3.3%) | 3 (3.2%) | 0.97 |
| Lymphogenous | 13 (52.0%) | 18 (18.2%) | **<0.001** | 13 (43.3%) | 18 (19.1%) | **0.008** |
| Local | 0 (0.0%) | 1 (1.0%) | 0.61 | 0 (0.0%) | 1 (1.1%) | 0.57 |

ITR, initial tumor reduction; PT, primary tumor; LN, lymph node.

**
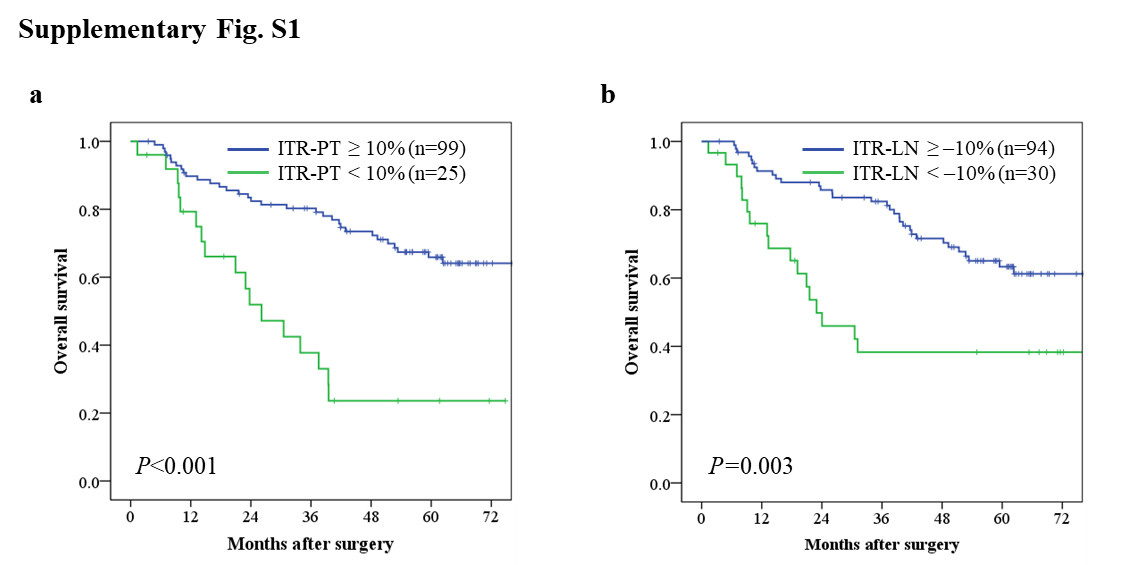
**

**FIGURE LEGENDS**

**Supplementary Fig. S1.** Kaplan–Meier overall survival according to (**a**) the initial tumor reduction of the primary tumor (ITR-PT) ≥10% and ITR-PT <10% groups and (**b**) the initial tumor reduction of the lymph node (ITR-LN) ≥–10% and the ITR-LN <–10% groups.
